# Atypical Sweet syndrome: skin sinus tracts in an acutely febrile patient after lymphoma treatment: a case report

**DOI:** 10.3389/fimmu.2023.1193808

**Published:** 2023-06-05

**Authors:** Shi-Ying Lu, Hui-Fang Yang, Qing-Li Zeng, Peng Chen, Li Chen, Jing Gao, Xue-Kui Gu, Hai Lan, Man Luo

**Affiliations:** ^1^ Department of Hematology, The Affiliated TCM Hospital of Guangzhou Medical University, Guangzhou, China; ^2^ Department of Hematology, The First Affiliated Hospital of Guangzhou University of Traditional Chinese Medicine, Guangzhou, China; ^3^ Department of HematologyShunde Hospital of Guangzhou University of Traditional Chinese Medicine, Guangzhou, China

**Keywords:** drug-induced Sweet syndrome, local sinus ulcer, systemic anaplastic large-cell lymphoma, granulocyte colony-stimulating factor, brentuximab vedotin

## Abstract

Sweet syndrome (SS) is an uncommon inflammatory disease that involves painful skin, edematous, red papules, plaques, or nodules often accompanied by fever and leukocytosis. SS has three subtypes, including classical, malignant-tumor associated, and drug-induced SS (DISS). Patients with DISS have clear histories of recent drug exposure. The incidence of SS is high in hematological malignancy but rare in lymphomas. Glucocorticoid treatment is the recommended treatment for all subtypes of SS. This case study describes a male patient who had a history of sALCL(Systemic anaplastic large cell lymphoma) and was treated with multiple cycles of monoclonal-antibody (mAb) therapy. They also received the G-CSF injection at the site where skin lesions later developed. They met the diagnosis criteria for DISS, which was considered to be caused by the G-CSF injection. In addition, BV(Brentuximab vedotin) administration might predispose them to DISS. This case illustrates the first reported SS during the lymphoma treatment, with rare clinical presentations of local crater-like suppurative skin lesions. This case expands the available literature on SS and hematologic neoplasms and reminds clinicians to promptly recognize and diagnose SS to minimize patient morbidity and long-term sequelae.

## 1 Introduction

Sweet syndrome (SS), or acute febrile neutrophilic dermatosis, is an uncommon inflammatory disorder characterized by the abrupt appearance of painful, edematous, and erythematous papules, plaques, nodules, or honeycomb-like or bullous changes on the skin. In addition, affected patients can have fever and leukocytosis, as well as eye, musculoskeletal, and internal organ involvement. SS was initially described by Dr. Sweet in 1964 (1). In 1971, the first cancer-related SS was reported (2). Later, drug-induced SS (DISS) was presented and its diagnostic criteria were proposed in 1986 (3). Researchers have since classified SS into three subtypes based on the etiologies, including classical, malignant-tumor associated, and DISS. Most SS subtypes can be directly distinguished by etiology.

Herein, we report on a case of SS that was considered to be related to two factors, including a history of lymphoma and anti-tumor drugs. The atypical clinical presentations of the skin sinus tracts in the patient made diagnosing SS challenging.

## 2 Case description

**Figure 1:**
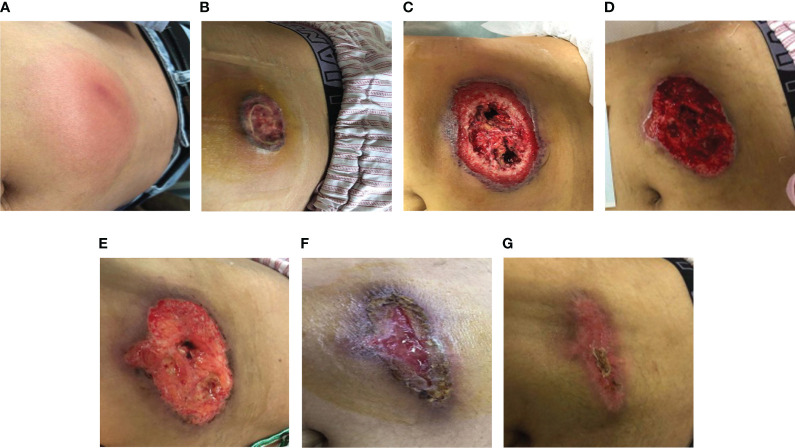
**(A)** The patient developed a subcutaneous induration in the abdominal wall. Redness, swelling, and pain worsened within 4 days. **(B)** Abdominal-wound tissue necrosis; ulcerated area: 2×2 cm. **(C)** Abdominal-ulcer worsened; ulcerated area: 10×10 cm. **(D)** The skin lesions had a poor response to the antibiotic treatment. **(E–G)**. After treatment with prednisone, the patient’s crater-like ulcer gradually healed.

A 30-year-old male patient was admitted to our hospital for 8 days with redness and swelling of the abdominal wall and left arm. The patient had systemic anaplastic large cell lymphoma (sALCL). He had received immune-chemotherapy with a BV-CHP regimen, including brentuximab vedotin (BV, 1.8 mg/kg on day 1), cyclophosphamide (750 mg/m^2^ on day 1), doxorubicin (50 mg/m^2^ on day 1), and prednisone (100 mg on days 1–5) (4) for four cycles and achieved complete remission (CR) during the second cycle. Ten days after his last treatment, the patient received granulocyte colony-stimulating factor (G-CSF 300 μg for 2 days) injections in the abdomen wall and left arm due to agranulocytosis. Seventy-two hours after G-CSF injections, the patient developed rapidly expanding erythema and induration in the areas of administration. In addition, he also reported low-grade fever. At hospital admission, the size of the abdominal wall induration had reached 10×10 cm^2^ (**Figure 1A**). The patient had no family history of neoplastic disease or severe dermatosis.

**Figure 2:**
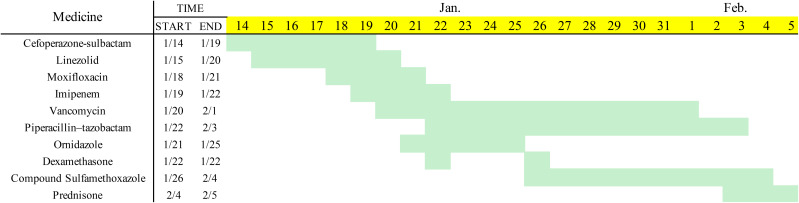
Record of drug use.

After admission, laboratory tests reported the following results: white blood cell count (WBC) 16.27×10^9^/L, neutrophil count 12.69×10^9^/L, lymphocyte count 0.89×10^9^/L, hemoglobin (Hgb) 113 g/L, platelet count (PLT) 257×10^9^/L, C-reactive protein (CRP) 139 mg/L, procalcitonin (PCT) 0.8 ng/mL, and serum amyloid A (SAA): >200 mg/L. Doppler ultrasound of the abdominal induration revealed swelling with limited fluid collection. The patient received antibiotic treatment with cefoperazone–sulbactam empirically. However, the skin erythema and induration gradually progressed to become ulcers (**Figure 1B**). His pain intensity increased and their body temperature also rose to >39°C, with chills. Antibiotic treatment was switched to linezolid, moxifloxacin, and topic mupirocin ointment. One week later, the repeat laboratory tests showed that CRP and PCT levels decreased to 77.2 mg/L and 0.57 ng/mL, respectively. However, the patient’s skin lesions continued to expand (**Figure 1C**). The ulcerated areas on his skin gradually developed abscesses. Antibiotics were then switched to imipenem, vancomycin, and ornidazole. However, the patient’s symptoms did not improve. He required frequent analgesics for pain and antipyretics for fever. We then further switched his antibiotics to piperacillin–tazobactam and sulfamethoxazole (**Figure 2**). During this process, wound secretions and blood were cultured several times. The tissue samples were also sent for metagenomic next-generation sequencing to detect potential pathogens. However, all these tests reported negative results. His skin lesions had a poor response to antibiotic treatment (**Figure 1D**).

**Figure 3:**
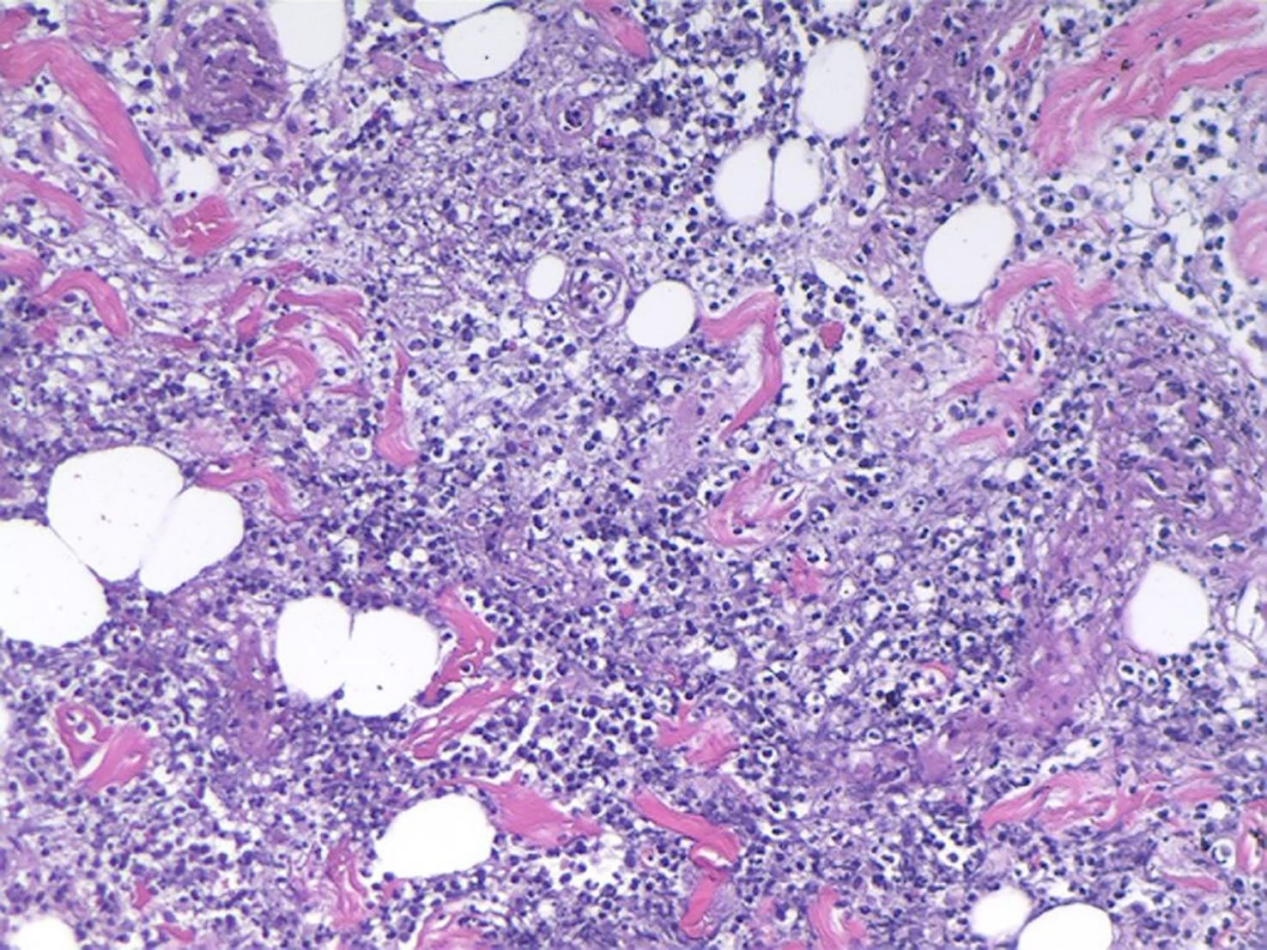
Muscle and adipose-tissue pathology results: diffuse and extensive neutrophil infiltrations.

**Figure 4:**
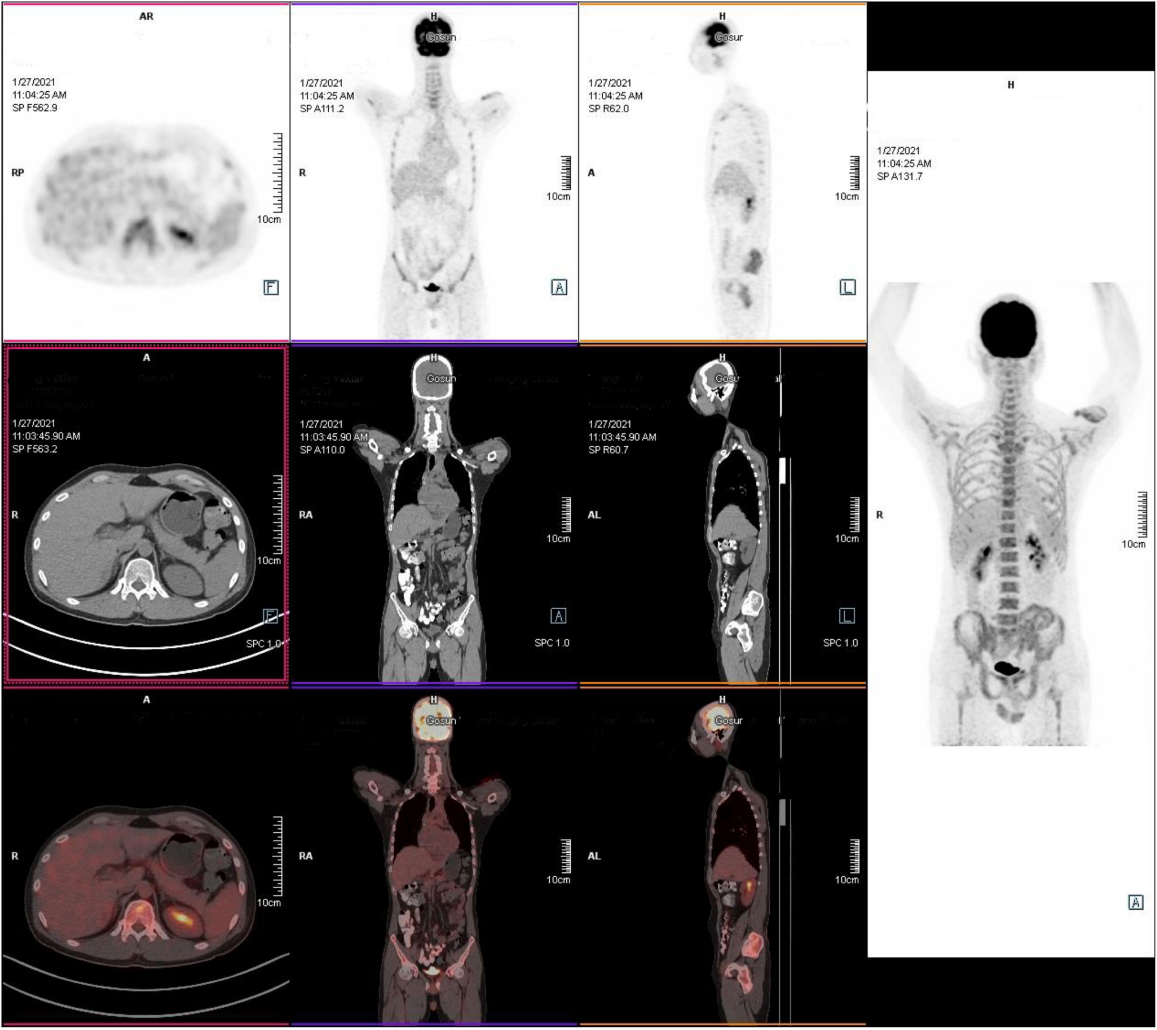
Positron emission tomography/computed tomography examination. The tumor was in remission without radiographic recurrence. (1) There was no increase in lesion metabolism in the original mediastinal, neck, axillar, supraclavicular, and internal mammary areas; the Deauville score was 1, which was considered complete remission after treatment. (2) Left upper-arm and lower-left abdominal-wall skin and subcutaneous inflammatory changes.

The patient had a history of lymphoma. Although the previous treatment had achieved CR, we considered possible tumor recurrence and skin invasion due to the poor response of skin lesions to the antibiotic treatments. We performed a skin lesion tissue biopsy. The pathological report showed diffuse neutrophil infiltrations in the muscle and adipose tissue without lymphoma cells (**Figure 3**). In addition, a positron emission tomography/computed tomography examination showed a negative result for lymphoma (**Figure 4**). Therefore, we ruled out tumor recurrence and skin invasion based on these two evaluations.

We then noticed that the patient had used two drugs (BV and G-CSF) before the development of the skin lesions. Therefore, we considered whether the skin lesions might be related to these two drugs.

After MDT(Multi-Disciplinary Treatment), combined with the patient’s current clinical signs and the wound’s pathological findings of massive neutrophil infiltrations, we considered the diagnosis of SS. According to his recent history of G-CSF injection and BV treatment, we speculated the subtype of DISS (5, 6) (diagnostic criteria shown in **Table 1**), since his presentation was consistent with a diagnosis of DISS based on the available clinical information. Therefore, we stopped all antibiotics and administrated glucocorticoid treatment 20 days after admission (prednisone, 40 mg q.d. initially and then gradually tapered and maintained for 1 month). After the initiation of prednisone, his temperature dropped rapidly, and the skin lesion steadily improved. On February 3, the patient’s body temperature returned to normal. The ulcer then gradually closed and the wound healed one month later (**Figures 1E–G**).

**Table 1:** Diagnostic criteria for drug-induced Sweet syndrome (DISS) (4, 5).

1. Abrupt onset of painful erythematous plaques or nodules
2. Histopathological evidence of dense neutrophilic infiltration without evidence of vasculitis
3. Pyrexia, >38°C
4. Temporal relationship between drug ingestion and clinical presentation, or temporally related recurrence after oral challenge
5. Temporally related resolution of lesions after drug withdrawal or treatment with systemic corticosteroids

Treatment results indicated that the patient was responsive to steroid therapy. This patient met all five diagnostic criteria for DISS (5, 6) (**Table 1**). Severe cutaneous-to-muscular sinus ulcers caused by SS had not been previously reported. These ulcers might be related to the history of sALCL and the G-CSF and BV use in this patient.

## 3 Discussion

SS induced by G-CSF has been reported in scientific literature (7, 8). The most commonly involved common skin wounds are papular lesions. Bullous ulcers are occasionally seen and can be complicated by pyoderma gangrenosum. SS is common in hematological malignancies. The inflammation is confined to the dermis. Our case’s unique features included rare sinus ulcers, a history of malignant tumors, and immunocompromised status from recent immuno-chemotherapy. It should also be noted that SS is a generalized cutaneous inflammatory lesion (9). Affected patients commonly have skin lesions all over the body. However, our patient only had skin wounds at the sites of G-CSF injection.

In this case, skin inflammation progressed rapidly, became necrotic, and quickly penetrated the subcutaneous and fatty tissue to form sinus tracts to the muscles. These SS skin lesions were atypical and difficult to distinguish from infection, which made clinical diagnosis and management difficult.

Meanwhile, ulcerative SS needs to be distinguished from Pyoderma Gangrenosum(PG). Both SS and PG belong to neutrophilic dermatosis. In the diagnosis of SS, the patient’s peripheral blood white blood cells and granulocytes can be significantly increased. At the time of admission, the patient had only honeycomb-like tissue changes of the skin, without ulcers, and significantly increased white blood cells and granulocytes in peripheral blood. The time from skin change to ulcer progression was fast in PG, while most SS only showed skin-like changes. The patient’s skin in our case took 11 days from local redness, redness, and swelling to rupture and form ulcers. The ulcer surface gradually expanded and deepened after formation, and the development of the ulcer surface slowed down after debridement. The skin did not form “wrinkled paper” changes after ulcers (10, 11) healing. Due to the specificity of the ulcer, in the later stage of this patient’s treatment, the clinical diagnosis was a special manifestation subtype of SS.

SS is a rare inflammatory disorder characterized by painful, edematous red papules, plaques, or nodules of the skin, often accompanied by fever and leukocytosis. It can also involve the eyes, muscles, bones, and internal organs (12, 13) (the diagnostic criteria are outlined in **Table 2**). SS is classified into three subtypes, classical SS, malignant-tumor–associated SS, and DISS (9).

**Table 2:** Major and minor diagnostic criteria for Sweet syndrome.

Criteria	Findings
Major	1. Abrupt onset of painful erythematous plaques or nodules
	2. Histopathological evidence of a dense neutrophilic infiltrate without evidence of leukocytoclastic vasculitis
Minor	1. Pyrexia, >38°C
	2. Association with an underlying hematological or visceral malignancy, inflammatory disease, or pregnancy; or preceded by an upper-respiratory or gastrointestinal inflection or vaccination
	3. Excellent response to treatment with systemic corticosteroids or potassium iodide
	4. Abnormal laboratory values at presentation (three or four): *Erythrocyte sedimentation rate (ESR) > 20 mm/h *White blood cell count (WBC) > 8×109/L *Neutrophil count > 70% *High C-reactive protein (CRP) levels

Classical SS is the main form of the syndrome and is associated with infection, inflammatory bowel disease, and pregnancy. It can be diagnosed only by ruling out malignancy and drug exposure (9, 12–14). SS associated with malignant tumors is mainly reported in patients with hematological malignancies (6, 15). The most common malignancy involved is acute myeloid leukemia, followed by myelodysplastic syndrome, Hodgkin lymphoma, and non-Hodgkin lymphoma (15, 16). The current literature suggests that SS can be a harbinger of impending malignancy and in some cases be the first sign of cancer recurrence (14).

The main characteristic of DISS is concurrence with drug use. It usually develops within 2 weeks after drug use (17). Repeated use of the same drug at the same site can cause DISS recurrence. G-CSF is currently thought to be the most common drug that induces DISS (18). Antitumor drugs can also lead to neutrophilic dermatosis (19, 20). To date, drugs that have been reported in DISS include azacitidine (21, 22), bortezomib (23, 24), imatinib (25–27), lenalidomide (19), and all-trans retinoic acid (28). With the increasing use of immune checkpoint inhibitors, it has been reported that SS could be induced by mAbs(Monoclonal antibodies) (29).

In our patient, it was unknown whether BV or CHP induced or predisposed him to DISS. BV has been reported to cause toxic necrosis of the skin and subcutaneous tissue. However, according to pharmacological studies of BV, the drug’s half-life is no more than 7 days. BV can cause epidermolytic poisoning, which often happens within 7 days (30). In our patient, the period from the last BV treatment and the initial onset of skin lesions was >10 days. The patient’s clinical manifestation was local skin ulceration, which was not consistent with the BV-induced skin lesions of generalized dermatitis. Therefore, we consider that his SS was less likely due to BV administration. The same consideration to the chemotherapy of CHP regimen, the most common adverse events are acute cystitis and myocardial cell injury. However, we could not exclude the possibility of BV as a predisposing factor. BV is an antibody drug conjugate, a combination of Cluster of Differentiation 30 (CD30)–directed antibody, and anti-tubulin monomethyl obestatin E (31) that is used as targeted immunotherapy against CD30 (32). Adverse skin reactions and subcutaneous-tissue diseases from BV have been reported, such as toxic epidermal necrolysis and Stevens–Johnson syndrome (33). In this case, multiple courses of BV treatments might cause increased immune activity that leads to hypersensitivity to the subsequent G-CSF injection.

Pathological studies indicate that CD30 can also be expressed in subcutaneous myoepithelial cells. This patient had a history of multiple BV injections, which might cause small amounts of BV immune complex accumulations in his subcutaneous tissue. After subcutaneous injection of G-CSF, the patient’s local tissues were stimulated to accumulate many neutrophils, which, together with the immune complex, triggered a local hypersensitivity reaction. Therefore, we could not rule out his severe ulcers as an Arthus reaction (34, 35).

Since the mechanism of SS is not fully understood, the dual effects of G-CSF and BV might have led to the co-existence of cytokine storm and hypersensitivity response, possibly causing severe sinus ulcerative SS in this patient.

## 4 Conclusion

This patient had a rare presentation of SS, characterized by hyperpyrexia and local sinus ulcers. These symptoms are not consistent with common systemic epidermal reaction in SS and are difficult to differentiate from local severe skin infections or tumor cell infiltrations. With the glucocorticoid therapy, his symptoms were relieved and the skin ulcers were fully healed. In this case, the onset of SS was associated with G-CSF injection. In addition, the skin lesions might have been related to his recent history of BV medication, which requires further investigation and attention during clinical practice.

## Data availability statement

The original contributions presented in the study are included in the article/supplementary material. Further inquiries can be directed to the corresponding author.

## Ethics statement

Written informed consent was obtained from the individual(s) for the publication of any potentially identifiable images or data included in this article.

## Author contributions

S-YL performed data analysis and drafted the manuscript. H-FY performed data analysis and prepared the manuscript. Q-LZ performed data analysis and prepared the manuscript. LC performed data analysis and prepared the manuscript. PC reviewed the article critically for important intellectual content. JG reviewed the article critically for important intellectual content. X-KG reviewed the article critically for important intellectual content. HL reviewed the article critically for important intellectual content. ML reviewed the article critically for important intellectual content. All authors contributed to the article and approved the submitted version.
